# Activity of tarloxotinib‐E in cells with *EGFR* exon‐20 insertion mutations and mechanisms of acquired resistance

**DOI:** 10.1111/1759-7714.13931

**Published:** 2021-03-12

**Authors:** Masaya Nishino, Kenichi Suda, Takamasa Koga, Shuta Ohara, Toshio Fujino, Junichi Soh, Vijaya Tirunagaru, Avanish Vellanki, Robert C. Doebele, Tetsuya Mitsudomi

**Affiliations:** ^1^ Division of Thoracic Surgery, Department of Surgery Kindai University Faculty of Medicine Osaka‐Sayama Japan; ^2^ Rain Therapeutics, Inc. Newark California USA

**Keywords:** *EGFR* mutation, lung cancer, molecular targeted therapy, acquired resistance, secondary mutations

## Abstract

**Background:**

Approximately 10% of non‐small cell lung cancers (NSCLCs) that harbor epidermal growth factor receptor (*EGFR*) gene mutations have in‐frame insertions in exon 20 of the *EGFR* gene. These tumors do not usually respond to currently available EGFR‐tyrosine kinase inhibitors (TKIs). Tarloxotinib is a novel hypoxia‐activated prodrug that releases a potent, irreversible pan‐ERBB TKI (tarloxotinib‐E) under solid tumor hypoxia.

**Methods:**

We examined the efficacy of tarloxotinib‐E against several types of Ba/F3 cells with introduced *EGFR* exon 20 mutations (*EGFR* A763insFQEA, V769insASV, D770insSVD, H773insH and H773insNPH mutations). We assayed growth inhibition for tarloxotinib (prodrug), tarloxotinib‐E (active form), poziotinib, afatinib, and osimertinib in Ba/F3 cells with each *EGFR* exon 20 mutation. We also explored acquired resistance mechanisms to tarloxotinib‐E by establishing cells with resistance to tarloxotinib‐E via chronic drug exposure after N‐ethyl‐N‐nitrosourea mutagenesis treatment.

**Results:**

Among all tested Ba/F3 cell lines, IC_50_ was ≥72.1 times higher for tarloxotinib than for tarloxotinib‐E, which implies a wide therapeutic window with this prodrug strategy. Tarloxotinib‐E was efficacious against all tested Ba/F3 cells except for H773insH, which was less sensitive to all tested EGFR‐TKIs. As acquired resistance mechanisms to tarloxotinib‐E, we identified either T790M or C797S secondary mutations, depending on the original *EGFR* exon 20 mutation.

**Conclusions:**

These findings indicate that tarloxotinib‐E could be effective for NSCLC with *EGFR* exon 20 mutations. Our results also show that T790M or C797S mutations can confer acquired resistance to tarloxotinib‐E; and suggest that resistance mechanisms are influenced by the baseline *EGFR* exon 20 mutations.

## Graphical abstract

Growth inhibitory curves for Ba/F3 cells with one of five most common EGFR exon 20 insertion mutations. Data are shown for tarloxotinib (inactive form), tarloxotinib‐E (activated form), afatinib, osimertinib, and poziotinib.

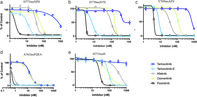

## INTRODUCTION

Epidermal growth factor receptor (EGFR) tyrosine kinase inhibitor (TKI) monotherapy is a standard of care for patients with recurrent or metastatic non‐small cell lung cancers (NSCLC) that harbor *EGFR* mutations, such as exon 19 deletion and L858R point mutation.^1^ However, approximately 10%–12% of *EGFR*‐mutated NSCLC tumors have an in‐frame insertion within exon 20 of *EGFR*.^2^ These tumors currently have no effective or approved targeted therapies. For example, response rates of first generation (1G) TKI erlotinib, 2G‐TKI afatinib, or 3G‐TKI osimertinib in patients with *EGFR* exon 20 mutations were reportedly 6%–11%.^3^, ^4^, ^5^ An irreversible, repurposed 2G EGFR‐TKI, poziotinib, reportedly has therapeutic potential for these tumors.^6^ However, because of its potent inhibition of wild‐type *EGFR*, severe skin and gastrointestinal adverse effects have been reported in clinical trials.^6^

Tarloxotinib is a hypoxia‐activated prodrug that is designed to release a potent and irreversible pan‐ERBB TKI (tarloxotinib‐E) under pathophysiological hypoxia (<0.1% O_2_), which is often present in solid tumors.^7^, ^8^ Briefly, one‐electron reduction of tarloxotinib under oxygen‐deficient conditions leads to loss of the trigger moiety, releasing the neutral, diffusible “warhead” tarloxotinib‐E. Mechanistic studies for this conversion show that tarloxotinib is cell‐excluded because of the positive charge of the quaternary ammonium cation to which the 4‐nitroimidazole trigger is appended. Anoxic conditions deplete cell‐associated tarloxotinib with a concomitant increase in the cell‐associated tarloxotinib‐E concentrations, which inhibit proliferation in various EGFR‐expressing cell lines. The geometry of tarloxotinib‐E release was spatially coordinated in vivo, with hypoxic regions in tumors from mice with SiHa tumor xenografts.^9^ In looking for molecules with important roles in this conversion, CRISPR/Cas9‐mediated disruption of the metalloreductase, STEAP4, showed it to be the dominant plasma membrane reductase that facilitates one‐electron reduction of tarloxotinib.^10^ We have reported that many lung tumors, including those with *EGFR* mutations, express STEAP4.^11^ Therefore, selective release of tarloxotinib‐E is expected to increase its dose intensity within tumors and enhance its tolerability, because of reduced inhibition of wild‐type *EGFR* in noncancerous tissues, compared with poziotinib and other EGFR‐TKIs. Tarloxotinib is currently in a Phase 2 clinical trial (NCT 03805841) for *EGFR* exon 20‐ and *HER2*‐activating mutations and other solid tumors with *NRG1/ERBB* gene fusions. First analysis of this clinical trial reported stable disease in 6/11 patients (55%) in the cohort of *EGFR* exon 20 insertions and tumor reduction by RECIST in 4/9 evaluable patients (44%) in the cohort of *HER2* activating mutation.^12^

This study investigated the efficacy of tarloxotinib‐E against Ba/F3 cells with *EGFR* exon 20 mutations. We selected the five most common *EGFR* exon 20 mutations in the COSMIC database (Table 1). In addition, we used the N‐ethyl‐N‐nitrosourea (ENU) mutagenesis technique to explore secondary mutations that might confer acquired resistance to tarloxotinib‐E in tumors with *EGFR* exon 20 mutations.

**TABLE 1:** Frequencies of *EGFR* exon 20 insertions in COSMIC database (searched on June 24, 2019)

Frequency of mutation subtypes		Numbers of reports	(%)
1	V769insASV	89	29.5
2	D770insSVD	61	20.2
3	H773insNPH	34	11.2
4	H773insH	18	6.0
5	A763insFQEA	16	5.3
	Others	84	27.8

## METHODS

### Cell culture, reagents, and establishment of Ba/F3 cells with EGFR^Exon20^

The interleukin‐3–dependent murine pro–B‐cell line Ba/F3 was provided by Riken Bio Resource Center. Cells were cultured in RPMI 1640 medium (Wako) with 10% FBS (Sigma‐Aldrich). EGFR‐TKIs (afatinib, poziotinib, and osimertinib) were purchased from Selleck Chemicals. Tarloxotinib and tarloxotinib‐E were provided by Rain Therapeutics, Inc. Mycoplasma contamination was checked routinely using the TaKaRa PCR Mycoplasma Detection Set (Takara).

Each *EGFR* exon 20 mutation was introduced into the Ba/F3 cells by retroviral vectors as previously described.^13^ We generated A763insFQEA, D770insSVD, and H773insNPH mutations from full‐length complementary DNA that encoded the wild‐type *EGFR* gene (Addgene), using the Prime STAR Mutagenesis Basal Kit (Takara) with specific primers (Table S1). Full‐length complementary DNA that encoded V769insASV and H773insH was purchased from Addgene. We also used Ba/F3 cells with secondary mutations, plus either *EGFR* exon 19 deletion or L858R point mutation, which our previous study showed to be potential factors in resistance to frontline osimertinib.^13^

### Growth inhibition assay

We seeded 2000 transfected Ba/F3 cells into each well of 96‐well plates, which were grown in RPMI 1640 medium supplemented with 10% FBS for 24 h. DMSO or an EGFR‐TKI at the indicated concentration was then added, and the cells were cultured for a further 72 h. We used colorimetric assays to estimate the growth inhibition of each drug using the Cell Counting Kit‐8 reagent (Dojindo Laboratories), following the manufacturer's protocol. Each experiment was performed in triplicate.

### Establishment of tarloxotinib‐E‐ or poziotinib‐resistant cells

Cells with acquired resistance against tarloxotinib‐E or poziotinib were established using the ENU (Sigma‐Aldrich) mutagenesis technique as previously described.^14^ ENU exposure was performed at the concentration of 100 μg/ml for 24 h. We seeded 5 × 10^4^ cells into 24 wells of 96‐well plates in the presence of tarloxotinib‐E (200 nM) or poziotinib (200 nM). These plates were incubated for two weeks with medium changes twice weekly; surviving clones were isolated. After establishing resistant cells, total DNA was extracted, and we explored secondary *EGFR* mutations between exons 18–21 by direct sequencing as previously described.^15^

## RESULTS

### Efficacy of tarloxotinib‐E against various *EGFR* exon 20 mutations

**FIGURE 1:**
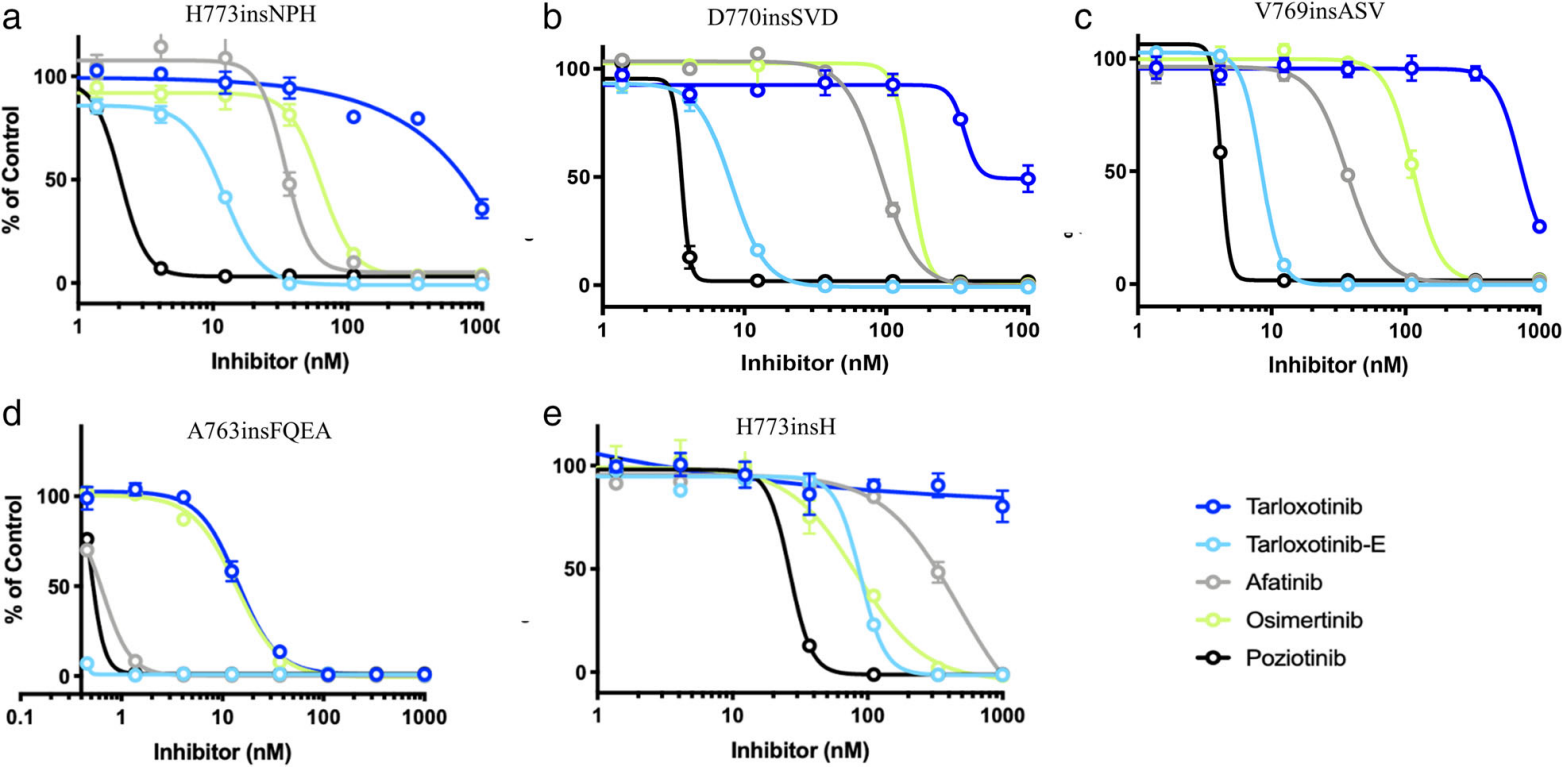
Growth inhibitory curves for Ba/F3 cells with various *EGFR* exon 20 insertion mutations. Data are shown for tarloxotinib (inactive form), tarloxotinib‐E (activated form), afatinib, osimertinib, and poziotinib in Ba/F3 cells with H773insNPH (a), D770insSVD (b), V769insASV (c), A763insFQEA (d), and H773insH (e)

Efficacies of afatinib, poziotinib, osimertinib, tarloxotinib, and tarloxotinib‐E were evaluated using cell growth inhibition assays (Figure 1, Table 2). Among all tested Ba/F3 cell lines, IC_50_ was ≥72.1 times higher for tarloxotinib than for tarloxotinib‐E, which implies a wide therapeutic window with this prodrug strategy. Using the cutoff value of 10 nM (50 nM for osimertinib because of its higher clinically achievable concentration), Ba/F3 cells with A763insFQEA showed sensitivity to all tested EGFR‐TKIs. Ba/F3 cells with V769insASV, H770insSVD, or H773insNPH were sensitive to tarloxotinib‐E and poziotinib, but not to afatinib or osimertinib. None of the tested EGFR‐TKIs showed strong efficacy against Ba/F3 cells with H773insH.

**TABLE 2:** Efficacy (IC_50_ values, nM) of each EGFR‐TKI for Ba/F3 cells that express *EGFR* exon 20 insertions

IC_50_ (nM)	Afatinib	Poziotinib	Osimertinib	Tarloxotinib	Tarloxotinib‐E
A763insFQEA	0.7	0.7	14.6	15.2	<0.5
V769insASV	35.5	4.8	118.4	675.9	7.6
D770insSVD	86.0	2.7	184.7	990.1	7.3
H773insNPH	35.8	2.2	61.9	714.0	9.9
H773insH	325	22.8	77.7	>1000	73.1

### Efficacy of tarloxotinib‐E against potentially osimertinib‐resistant cells

We also evaluated the efficacy of tarloxotinib and tarloxotinib‐E in Ba/F3 cells with potential resistance‐conferring mutations that may arise after front‐line osimertinib treatment.^13^ In our previous study, we established Ba/F3 cells with *EGFR* exon 19 deletions together with one of L718V, G724S, L792F, L792H, or C797S mutations; and Ba/F3 cells with L858R point mutation together with one of L718Q, L718V, L792F, L792H, C797G, or C797S mutations. Tarloxotinib‐E showed quite low IC_50_ values against these Ba/F3 cells, except for those with L858R plus C797S (Table 3). However, tarloxotinib‐E was ineffective against triple mutations, such as *EGFR* exon 19 deletion or L858R mutation plus T790M plus C797S, which could arise as an acquired resistance mechanism to osimertinib in a second‐line setting after 1G or 2G EGFR‐TKI therapy.

**TABLE 3:** Efficacy (IC_50_ values, nM) of each EGFR‐TKI for Ba/F3 cells with secondary mutations that may confer resistance to osimertinib

Type of *EGFR* mutation	Afatinib^12^	Osimertinib^12^	Tarloxotinib	Tarloxotinib‐E
Del19 + L718V	0.5	10.6	46.7	0.68
Del19 + G724S	<0.5	3.79	1.1	<0.5
Del19 + L792F	<0.5	4.01	16.9	<0.5
Del19 + L792H	0.6	11.9	0.6	<0.5
Del19 + C797S	2.8	791.4	408.2	5.1
L858R + L718Q	3.37	533.0	190.1	1.9
L858R + L718V	0.7	167.9	41.3	<0.5
L858R + L792F	0.97	29.5	189.9	<0.5
L858R + L792H	1.25	44.7	6.5	7.8
L858R + C797G	1.38	561.2	465.3	<0.5
L858R + C797S	6.68	918.0	>1000	54.2

### Secondary resistance mutations after tarloxotinib‐E treatment

We also explored acquired resistance mechanisms to tarloxotinib‐E in Ba/F3 cells with *EGFR* exon 20 mutations. After chronic exposure to tarloxotinib‐E, we acquired 62 tarloxotinib‐E‐resistant clones. We also established 57 poziotinib‐resistant clones, to confirm results after ending the tarloxotinib‐E experiments. The A763insFQEA cells were highly sensitive to tarloxotinib‐E and only one resistant clone (with acquired C797S) was obtained. In Ba/F3 cells with V769insASV, all 14 tarloxotinib‐E‐resistant clones and all 15 poziotinib‐resistant clones developed a T790M mutation. In Ba/F3 cells with D770insSVD, all 24 tarloxotinib‐E‐resistant clones and 20 poziotinib‐resistant clones developed a C797S mutation. In Ba/F3 cells with H773insNPH, 22 of 23 tarloxotinib‐E‐resistant clones and 21 of 22 poziotinib‐resistant clones developed a T790M mutation; the other clones harbored a C797S mutation. The growth inhibitory assay showed that the IC_50_ values of these resistant cells were >100 times higher than parental cells, and these cells were also insensitive to 2G‐TKI afatinib and 3G‐TKI osimertinib (Table 4). We introduced a C797S secondary mutation into Ba/F3 cells with V769insASV, which developed a T790M mutation through chronic drug exposure. We also introduced a T790M secondary mutation into Ba/F3 cells with D770insSVD, which developed a C797S mutation through chronic drug exposure. These cells were also insensitive to tarloxotinib‐E or poziotinib (Table 4).

**TABLE 4:** IC_50_ values of EGFR‐TKIs in Ba/F3 cells with both *EGFR* exon 20 insertions and a secondary mutation

Type of *EGFR* mutation		Afatinib	Poziotinib	Osimertinib	Tarloxotinib	Tarloxotinib‐E
A763insFQEA	+ T790M^a^	17.4	13.4	30.0	547.6	5.6
	+ C797S	45.2	17.7	959.2	>1000	186.8
V769insASV	+ T790M	415.1	263.4	55.1	>1000	104.3
	+ C797S^a^	985.2	619.7	>1000	>1000	>1000
D770insSVD	+ T790M^a^	741.1	214.6	54.1	>1000	471.7
	+ C797S	696.3	409.8	>1000	>1000	507.9
H773insNPH	+ T790M	>1000	534.9	107.3	>1000	496.6
	+ C797S	>1000	>1000	>1000	>1000	>1000

^a^ These cells were established by transfection of mutant *EGFR*. Others were established via chronic drug exposure after ENU mutagenesis.

## DISCUSSION

Development of effective therapy for NSCLC with *EGFR* exon 20 mutation is an unmet clinical need. Some rare *EGFR* exon 20 mutations are sensitive to currently available EGFR‐TKIs; e.g., A763insFQEA was sensitive to erlotinib^16^ and D770delinsGY showed partial response to an irreversible EGFR‐TKI, dacomitinib.^17^ However, currently available EGFR‐TKIs usually cannot inhibit *EGFR* exon 20 mutations. Many *EGFR* exon 20 mutation variants are reported in the COSMIC database. In this study, we established Ba/F3 cells with five most common *EGFR* exon 20 mutations, including the aforementioned A763insFQEA mutation, which was sensitive to erlotinib. These five variants accounted for 72.2% of all recurrent *EGFR* exon 20 mutations in the COSMIC database (Table 1).

As expected, A763insFQEA was sensitive to all tested EGFR‐TKIs including tarloxotinib‐E and poziotinib. Our results also showed that V769insASV, D770insSVD, and H773insNPH were sensitive to both tarloxotinib‐E and poziotinib. These results suggest that tarloxotinib‐E and poziotinib may be effective against many *EGFR* exon 20 mutations. Therefore, if conversion from tarloxotinib to tarloxotinib‐E in tumor tissues of human subjects is confirmed, tarloxotinib might be used to treat lung cancers with *EGFR* exon 20 mutations.

However, we found that the fourth most common *EGFR* exon 20 mutation, H773insH, did not respond well to either tarloxotinib‐E or poziotinib. We also showed that tarloxotinib‐E and poziotinib were not active against so‐called triple mutations, such as exon 19 deletion or L858R plus T790M plus C797S mutations, which can arise after 1G/2G EGFR‐TKI and osimertinib treatment failures. As tarloxotinib‐E and poziotinib are quite potent irreversible EGFR‐TKIs, another strategy, such as combination of an allosteric inhibitor and a quinazoline‐based EGFR‐TKI, will be needed to inhibit these resistant *EGFR* mutants.^18^ Notably, however, tarloxotinib‐E showed quite low IC_50_ values against Ba/F3 cells with secondary mutations that may confer acquired resistance to front‐line osimertinib.^13^ Osimertinib was approved as a first‐line treatment for lung cancer with *EGFR* mutations in 2018, and therefore tarloxotinib may be a suitable second‐line EGFR‐TKI for lung cancers with exon 19 deletions or with L858R point mutation.

In analyzing the potential resistance mechanisms to tarloxotinib‐E and poziotinib, we found that the acquisition of a T790M or C797S mutation to a *EGFR* exon 20 mutation was enough to confer resistance to these drugs (Table 4). Additionally, we observed some preference for specific secondary mutation in each type of *EGFR* exon 20 mutation (T790M for V769insASV and H773insNPH, and C797S for D770insSVD) in the drug exposure experiments. Although the mechanism for these preferences is unclear, the aforementioned study^17^ also observed that only T790M developed after chronic dacomitinib exposure in D770delinsGY Ba/F3 cells, whereas Ba/F3 cells that contained either T790M or C797S together with D770delinsGY were resistant to dacomitinib and afatinib. Similarly, *EGFR* with an exon 19 deletion—but not *EGFR* L858R—reportedly developed resistance to the 3G inhibitor osimertinib via L724S, which further supports the notion that resistance mutations are influenced by baseline mutations.^19^

In conclusion, our results indicate that tarloxotinib‐E could be effective for most NSCLCs with *EGFR* exon 20 mutations, or those for which front‐line osimertinib treatment is no longer effective. However, continuous efforts to develop novel EGFR inhibitors are necessary, as some *EGFR* mutants, such as D770insH or *EGFR* exon 20 mutation plus T790M or C797S, are refractory to tarloxotinib‐E and poziotinib in vitro.

## CONFLICT OF INTEREST

Dr Suda has received honoraria from Boehringer Ingelheim, has been a consultant/advisory board member for AstraZeneca, and has received research funding (through Kindai University Faculty of Medicine) from Rain Therapeutics and Boehringer Ingelheim. Mr Vellanki is currently an employee of Rain Therapeutics and owns stock in Rain Therapeutics. Dr Tirunagaru is currently an employee of Rain Therapeutics and owns stock in Rain Therapeutics. Dr Doebele is currently an employee of Rain Therapeutics and owns stock in Rain Therapeutics. Dr Doebele has received personal fees from Genentech/Roche, Ignyta, Blueprint Medicines, Green Peptide, AstraZeneca, Anchiano, Takeda/Millenium, and Bayer outside the submitted work; in addition, Dr Doebele has a patent for U.S. Provisional Patent Application No. 62/712531 pending and licensed to Rain Therapeutics; and The University of Colorado has received licensing fees from Foundation Medicine, Ignyta, Scorpion Therapeutics, Voronoi, Pearl River, Black Diamond Therapeutics, and Genentech for biological materials derived in Dr Doebele's laboratory. Dr Mitsudomi has received honoraria from AstraZeneca, Boehringer Ingelheim, Chugai, and Pfizer, has been a consultant/advisory board member for AstraZeneca, Chugai, and Boehringer Ingelheim, and has received research funding (through Kindai University Faculty of Medicine) from AstraZeneca, Boehringer Ingelheim, and Chugai. The other authors declare no conflicts of interest.

## Supporting information


**Table S1**. List of primers for mutagenesis PCR.
