# Supplementary material for: Activity of tarloxotinib‐E in cells with EGFR exon‐20 insertion mutations and mechanisms of acquired resistance
Source: Thorac Cancer. 2021 Mar 12;12(10):1511–6. doi: 10.1111/1759-7714.13931 (PMC8107039; doi:10.1111/1759-7714.13931)
Supplement: Supplementary file 1 — Table S1. List of primers for mutagenesis PCR. [file TCA-12-1511-s001.pdf]

| Supplementary Table 1. List of primers for mutagenesis PCR | | |
|---|---|---|
| | forward primer | reverse primer |
| A763insFQEA | 5’-CTTCCAGGAAGCCTACGTGATGGCCAGCGTG-3’ | 5’-CGTAGGCTTCCTGGAAGGCTTCATCGAGGATTT-3’ |
| D770insSVD | 5’-GACAGCGTGGACAACCCCCACGTGTGCCG-3’ | 5’-GGGTTGTCCACGCTGTCCACGCTGGCCATC-3’ |
| H773insNPH | 5’-CCACAACCCCCACGTGTGCCGCCTGCTGGGCA-3’ | 5’-ACGTGGGGGTTGTGGGGGTTGTCCACGCTGG -3’ |
| T790M | 5'-ACTCATCATGCAGCTCATGCCCTTCGG-3’ | 5’-AGCTGCATGATGAGTTGCACGGTGGAGG-3' |
| C797S | 5’-CCTTCGGCAGCCTCCTGGACTATGTCCGG-3’ | 5’-GGAGGCTGCCGAAGGGCATGAGCTGCG -3’ |
